# LONELINESS, MARITAL STATUS, AND COGNITIVE IMPAIRMENT AMONG OLDER AMERICANS

**DOI:** 10.1093/geroni/igz038.1387

**Published:** 2019-11-08

**Authors:** Jack Lam, Anthony R Bardo, Takashi Yamashita

**Affiliations:** 1 University of Queensland, Brisbane, Queensland, Australia; 2 University of Kentucky, Lexington, Kentucky, United States; 3 University of Maryland, Baltimore County, Baltimore, Maryland, United States

## Abstract

Loneliness has been linked to increased risk of mortality and morbidity, and emergent research has identified a negative association between loneliness and cognitive functioning. While the determinants of loneliness are wide in scope, loneliness is closely tied to marital status in later life. At the same time, research has shown that those who are married have lower risk of cognitive impairment. The aim of this study was to determine the association between loneliness and cognitive impairment, and examine whether it is moderated by marital status. Data come from 9 waves of the RAND version of the HRS (1998 - 2014). Consistent with previous research, results from random effects logit models showed that loneliness is associated with greater risk of cognitive impairment [Odds-ratio (OR) = 1.41, p < 0.01]. Additionally, those who are widowed (OR = 1.29, p < 0.01), separated/divorced (OR = 1.33, p < 0.01), or never married (OR = 1.70, p < 0.01) are also more likely to have a cognitive impairment, compared to those who are married. However, the association between loneliness and cognitive function was found to only differ among those who are widowed. Contrary to expectations, widows who report feeling lonely are 29% (p < 0.01) less likely to have a cognitive impairment. In sum, while loneliness and marital status are closely linked with one another, they are both independent determinants of cognitive impairment. The distinct theoretical mechanisms linking loneliness and marital status to cognitive function in later life are discussed.

